# Exploring local realities: Perceptions and experiences of healthcare workers on the management and control of drug-resistant tuberculosis in Addis Ababa, Ethiopia

**DOI:** 10.1371/journal.pone.0224277

**Published:** 2019-11-13

**Authors:** Kirubel Manyazewal Mussie, Solomon Abebe Yimer, Tsegahun Manyazewal, Christoph Gradmann

**Affiliations:** 1 Institute of Health and Society, University of Oslo, Oslo, Norway; 2 Department of Microbiology, Unit for Genome Dynamics, University of Oslo, Oslo, Norway; 3 Coalition for Epidemic Preparedness Innovations, Oslo, Norway; 4 Addis Ababa University, College of Health Sciences, Center for Innovative Drug Development and Therapeutic Trials for Africa, Addis Ababa, Ethiopia; University of Ghana College of Health Sciences, GHANA

## Abstract

**Background:**

Drug-resistant tuberculosis (DR-TB) remains a major health security threat worldwide. The effectiveness of implementation of DR-TB control strategies has been a subject of research and controversy. In resource-limited settings, using conventional medicine as the only framework to explain DR-TB gives a rather incomplete picture of the disease. This study intended to explore the perceptions and experiences of healthcare workers on the management and control of DR-TB in Addis Ababa, Ethiopia.

**Methods:**

The study employed a qualitative methodology with an inductive approach and a thematic analysis. It involved in-depth interviews with healthcare workers providing clinical services to DR-TB patients in 10 public healthcare facilities in Addis Ababa, Ethiopia.

**Results:**

A total of 18 healthcare workers participated until data saturation, which included 12 clinical nurses, four health officers and two medical laboratory technicians. The findings show that healthcare workers perceive DR-TB as a growing public health threat in Ethiopia, due to factors such as poverty, poor nutrition, crowded settings, healthcare worker and general public awareness of DR-TB, lack of good governance and culture.

**Conclusion:**

The perspectives drawn from the healthcare workers shed more light on the image of DR-TB in a developing country context. It has been shown that understanding DR-TB is not confined to what can be drawn from the sphere of biomedicine. There are also interconnected barriers, which predict a dystopia in the epidemiology of DR-TB. Bringing DR-TB under control requires taking a step back from an overwhelming focus on the biomedical facets of the disease, and employ critical thinking on the wider social and structural forces as equally important targets.

## Introduction

According to literature, acknowledging the association between tuberculosis (TB) and poverty is admitting the reality of the disease and the social world in which patients live [1–4]. The attachment between TB and poverty has its roots in history, with the bond between the two remaining strong over time. Poverty contributes to the supply of poor quality drugs, poor adherence among TB patients and incorrect prescriptions by healthcare workers. These favor the development of drug-resistant TB (DR-TB) – TB that develops resistance to the antimicrobial drugs used to cure the disease. DR-TB remains a public health crisis and a major health security threat worldwide [5,6]. According to the WHO’s 2018 report [7], there were an estimated 558,000 new DR-TB cases in the reporting year, with only 55% of patients successfully treated.

Being a resource-poor continent, Africa contributes a quarter of new TB cases and TB deaths worldwide, with 2.5 million people falling ill and 417,000 people dying from TB annually [7]. Ethiopia, where this study was conducted, is among the countries with a high burden [8] and transmission potential [9] of DR-TB. In the current situation in which Ethiopia is struggling to overcome TB, the unprecedented prevalence of DR-TB is hindering efforts. The WHO reported that 172,000 people fell ill with-, and 29,000 died of, TB in 2017, while in the same year 5,500 people fell ill with DR-TB [7]. These mirror the setbacks of Africa in general, and Ethiopia in particular, to meet the WHO End TB strategy targets by 2035 [10] unless local realities – lived and subjective experiences as expressed by the bearers themselves – are explored to understand and effectively address the gaps.

The effectiveness of the implementation of DR-TB strategies in resource-limited settings, such as many areas of Sub-Saharan Africa, has been a subject of research and controversy for many years [11–13]. Most of the research output emerged from the traditional biomedical perspective that is more attentive to quantifiable and experimental objective explanations. By contrast, subjective experiences and perceptions reflecting perspectives of TB program implementers, such as healthcare workers, have hardly been explored.

Healthcare workers’ perspectives are essential, as they represent the grassroots efforts to manage and control TB in general. Because they meet the public on a daily basis, healthcare workers play a vital role, not merely in the betterment of DR-TB healthcare services, but also in developing and achieving TB strategic outcomes and translating DR-TB strategies into action [14,15]. On the other side, it is easy to anticipate that working in healthcare facilities, particularly having frequent and direct contact with DR-TB patients, increases the risk of infection among healthcare workers [16,17]. Cultural, social and economic factors that surround healthcare workers’ working environment affect their role in DR-TB management and control [18]. Their optimal understanding of-, and experience in, DR-TB and its treatment is important evidence in helping to shape the epidemiology of the disease and to achieve the goal of universal access to DR-TB care and services [19]. Hence, the learning process healthcare workers pass through influences and shapes how they perceive their work as DR-TB care providers.

Understanding these realities, the WHO developed a framework to engage healthcare providers in the management of DR-TB [20]. This framework describes approaches to engage different healthcare providers and other stakeholders in the various conditions of DR-TB care and management. The framework emphasizes the need for learning from the perceptions and experiences of individual healthcare workers in the management and control of TB, which is in line with this study.

In Ethiopia, little is documented on the perception and experience of healthcare workers on DR-TB management and control. The available literature mainly focuses on drug-sensitive TB [21–26], while DR-TB is poorly explored. Thus, to fill this gap, this study explored the perceptions and experiences of healthcare workers in the management and control of DR-TB in Addis Ababa, Ethiopia.

## Methods

### Design

Given that the aim is to explore perception and experience, this study uses qualitative methods. More specifically, it followed an inductive approach to generate meanings from the data in order to identify patterns and relationships of themes and concepts.

### Setting

The research was undertaken in Addis Ababa, which is the capital and one of the 11 administrative divisions of Ethiopia. The city is divided into 10 sub-city administrations, with each having 10-15 suburbs (“Woreda” in the local language).

The study included 10 public healthcare facilities distributed throughout the city, among which eight are sub-city level public healthcare centers accountable to the Addis Ababa City Health Bureau, whereas the remaining two are TB-specialized hospitals accountable to the Federal Ministry of Health (FMoH). The two TB specialized hospitals were purposively selected because they are the only referral hospitals in the entire city that diagnose and admit DR-TB patients. The number of the remaining eight healthcare centers was determined by the level of data saturation – in qualitative research, when new ideas cease to emerge in the data [27,28].

### Participants

The total number of participants in the study was 18. We used data saturation criteria to determine the required number of participants, with the study participants being purposively selected from 10 public healthcare facilities. The selection of participants from every healthcare facility was also facilitated by medical directors. The inclusion criteria were being more than 18 years old, being an employee in a healthcare facility, having worked and/or currently working with DR-TB patients, having studied one of the health science disciplines at a university/college on the diploma (10+3) or undergraduate degree level and above, and being willing to participate. The exclusion criteria were being below the age of 18, having never worked with TB patients and being unwilling to participate.

### Sampling process

According to the legal procedure in the healthcare facilities, a medical director at each healthcare facility should first give verbal approval for a researcher to proceed to contact a participant. Therefore, after getting an approval letter for the study from the Addis Ababa Health Bureau (further details under “Ethics”), we contacted a medical director at each healthcare facility to select the participants. All of the medical directors received the letters and gave their verbal approval. Some recruited the participants themselves based on the information they read in the letter, whereas others allowed us to select by ourselves. At the end of this, the eligible participants agreed and joined the study.

### Data collection

We considered in-depth interviews to be the most appropriate to explore the research topic. The fieldwork was conducted between August and October 2017, with all the interviews conducted in private rooms during work hours. The participants themselves scheduled when and where they were to be interviewed, and reported that they were comfortable about their decisions.

The language of communication with the participants during the in-depth interviews was Amharic, which is the mother tongue of the first, second and third authors (KMM, SAY, TM). As far as the interviewer (the primary investigator) noticed, none of the participants experienced difficulties in using this language. The absence of a language barrier was one contributing factor that made the interviews with the participants clear and lively.

The primary investigator conducted an in-depth interview with one healthcare worker to test the validity of the interview questions and gain experience in conducting an interview. Neither the participant nor the findings of the pretesting were used for the study.

The methodology of this research was designed to provide a thick description of concepts through interactive and open discussions with the participants. The primary investigator conducted in-depth interviews with all participants, took some notes and audio-recorded the interviews for later transcription. On average, each in-depth interview took 45-55 minutes. The use of audio records depended on the participants’ consent, and each of them was asked whether they were comfortable with that. All agreed to be recorded with the exception of one healthcare worker who requested the investigator to take notes while he/she spoke slowly.

### Data management and analysis

The investigators started analyzing the material during their fieldwork. This was done to identify gaps or a lack of information and, therefore, to produce a thicker description of topics through the interviews. The audio files from the in-depth interviews were then transcribed using Amharic, which produced a 97-page MS word document. The data analysis framework we used was Braun and Clark’s reflexive thematic analysis framework, and the analysis process followed the six phases this framework suggests – familiarization, initial coding, theme construction, reviewing themes, defining themes and producing the report [29]. The data was manually and openly coded line-by-line using MS word. This produced 95 codes – words and abbreviations used to represent ideas in the material. Having this number of codes helped to break down the data into meaningful segments without reducing them. The investigators repeatedly went through the entire material and looked at the codes to search for similarities among them, then developed broad themes that kept the data’s context. These themes differed from one another in terms of scope; some of them were broader, while others were narrower and fell under the broad themes.

The language of analysis was a mixture of both English and Amharic, meaning that the entire transcription was not translated into English. At the initial stage of writing, the findings, the themes and their description were written in English, whereas quotes taken from the data to support the themes stayed in the original language, Amharic. When the findings’ write-up was completed, the Amharic quotes were translated into English.

### Trustworthiness

The study followed certain criteria to ensure that the findings are trustworthy. The participants were selected based on criteria that ensure their eligibility to provide credible information. In addition, other colleagues participated to examine the translation of texts and methodology, and to identify any biases or errors in the study. In order to facilitate the transferability of the findings to other contexts, the details of the entire research process were recorded and all necessary documents kept so that the audience or other researchers could understand how the data was gathered and analyzed. In order to establish the dependability of the data, the authors have given a detailed description of the research methodology. A code-recoding procedure was applied in order to see any differences, and to ensure the confirmability of findings, each step from data collection through analysis is presented in detail. The tools used to collect data (both in English and Amharic) are annexed with this article (Annex I). Moreover, the data collected during fieldwork was discussed with some qualitative research professors during methodology seminars, as well as with fellow students and research supervisors.

### Ethical considerations

This research was first approved by the Norwegian Center for Research Data in Norway. In Ethiopia, ethical clearance was obtained from the Addis Ababa City Administration Health Bureau, and two separate ethical clearances were obtained from two federal hospitals specializing in TB. All the participants provided their written informed consent, and their anonymity and confidentiality were guaranteed by using fictitious names instead of their real names. Audio files were kept in safe storage, where the primary investigator was the only person who had access, and were deleted at the end of the study.

## Results

The findings resulted from an analysis of data collected from 18 healthcare workers at eight primary healthcare centers and two hospitals specializing in TB in Addis Ababa, Ethiopia (see Table 1). This was done through 18 in-depth interviews, with no major discrepancy found in the perceptions of the participants on the management and control of DR-TB. The analysis resulted in two themes and eight categories (see Table 2), which are illustrated with quotes:

**Table 1:** Demographic characteristics of study participants.

Name (anonymized)	Sex	Education level	Profession/title	Total service period	TB-specific service period
Kotno	Female	BSc Nursing	Clinical nurse	1 year	1 year
Hansir	Female	BSc Nursing	Clinical nurse	1 year	1 year
Kofi	Female	BSc Medical laboratory	Lab technologist	2 and a half years	3 months
Kasge	Male	BSc Public Health	Health officer	9 years	2 and a half years
Lidmu	Male	BSc Public Health	Health Officer	16 years	1 and a half years
Melsa	Male	BSc Nursing	Clinical nurse	4 years	2 months
Teman	Male	BSc Public Health	Health Officer	9 years	2 years
Kash	Female	BSc Nursing	Clinical nurse	3 months	3 months
Petol	Male	BSc Nursing	Clinical nurse	34 years	29 years
Pesar	Female	BSc Nursing	Clinical nurse	1 year	3 months
Mapet	Male	BSc Nursing	Clinical nurse	1 year	2 months
Pelas	Male	BSc Nursing	Clinical nurse	1 year	5 months
Shalpet	Female	BSc Nursing	Clinical nurse	1 year	1 year
Algon	Female	BSc Public Health	Health Officer	5 years	6 months
Alto	Male	BSc Nursing	Clinical nurse	1 year	6 months
Treal	Male	Diploma Nursing	Clinical nurse	2 years	2 months
Almet	Male	BSC Medical laboratory	Lab technologist	5 years	1 year
Ambal	Male	BSc Nursing	Clinical nurse	2 years	3 months

**Table 2:** List of the themes and sub-themes that resulted from the data analysis.

Themes	Sub-themes/Categories
**Theme 1** Workers’ perceptions of the current state of DR-TB and prediction of its future	1.1. Chances of overcoming DR-TB 1.2. DR-TB as a disease of poverty 1.3. Poor nutrition 1.4. Crowded home settings 1.5. Movement of clients
**Theme 2** Experiences of management and control of DR-TB	2.1 Public awareness of DR-TB 2.2 Healthcare workers’ clinical knowledge on DR-TB 2.3 Cultural values among clients

### Theme 1: Workers’ perceptions of the current state of DR-TB and the prediction of its future

This theme illustrates the healthcare providers’ perceptions on the current state of DR-TB. Moreover, it presents what they anticipate based on the current perceived seriousness of the disease. This theme and its five subthemes were related to the current state of DR-TB and the prediction of its future. The subthemes supported by descriptions and quotes, are as follows.

#### Chances of overcoming DR-TB

All healthcare workers perceive that the problem of DR-TB is getting worse as time goes by. The healthcare workers perceive DR-TB as a disease that will continue to be a public health threat. The participants indicated that the trend of TB is moving, and will continue to move, towards more DR-TB cases than before. Exacerbated by socioeconomic factors, DR-TB will worsen and be beyond the capacities of current control efforts in the country. According to the participants, this manifested itself through the current tragic conditions in healthcare centers that could not cope up with the growing number of DR-TB patients.

“*I have no words to tell you how serious the problem is*. *Soon*, *it will be difficult to detect active TB in the community because we will have too much of XDR (extensively drug-resistant) and MDR (multidrug-resistant) -TB instead*. *…It is not as simple as you and me talking about it here*.*” (Lidmu*, *health officer)*

“*There is no light that makes us think drug-resistant TB is decreasing*.*” (Teman*, *health officer)*

“*u u u …* [this is translated from the Amharic letters “ኡኡኡ ….” and it is a sound an Ethiopian makes when hearing (extremely) bad news – death of a relative or something unexpected] *if it continues like this*, *I am afraid everyone will be infected and there will remain no one to treat*! *…I think the problem is becoming out of control and our capacity*.*” (Ambal*, *clinical nurse)*

#### DR-TB as a disease of poverty

All of the participants reported one issue, which in their opinion plays a dominant role – poverty. For all of the healthcare workers, poverty is to blame in the general epidemiology of DR-TB. This disease is a problem for poor people, of those who work under unfavorable conditions and who are economically weak. The participants mentioned some of the ways – living conditions and nutrition, for example – in which poverty plays a role in the epidemiology of DR-TB. These are further discussed in the subsequent subthemes. In addition to being linked to a person's economic strength, DR-TB is related to lifestyle in general; the way people live also comes into play. Transportation problems, malnutrition and financial weakness, to mention just some, are the ways in which poverty imposes its power over people. The participants argued that the fact that DR-TB troubles developing countries like Ethiopia, but not the developed world, is evidence that it is a disease of poverty. Poverty contributes to the growth of DR-TB, not only by incapacitating health systems, but also by making people more vulnerable to the disease.

“*It is true that TB is called a disease of poverty*. *… It is a disease of poverty*, *period*!*” (Hansir*, *clinical nurse)*

“*TB in general is mainly a problem of developing countries*, *not of the developed ones*.*” (Petol*, *clinical nurse)*

“*It would be good if everyone could protect themselves (from DR-TB)*. *But we cannot do this*, *…*.*because of poverty (silence and tears followed)*.*” (Algon*, *health officer)*

#### Poor nutrition

This study finds that from the healthcare workers’ perception and experience, patients are facing challenges in adhering to treatment due to a poor diet and the inability to cover the financial crises created by treatments that take time. Given the fact that being on second-line drugs is a hard life and demands that the person eats well, the absence of a nutritious diet due to poverty makes adhering to the treatment very difficult for the patient. Good nutrition is not only related to treatment after being a patient, but also to protecting oneself from the disease – a rich diet helps build the immune system, which further reduces the risk of falling ill from TB and its complications (drug resistance).

“*The drugs are taken for a long time so they need you to eat good food*.*” (Hansir*, *clinical nurse)*

“*Nutrition plays a big role*. *If there is no protein in you then the drug alone does not work*.*” (Kasge*, *health officer)*

#### Crowded home settings

The participants stated that DR-TB relates to living in small rooms as a big and extended family.

“*I remember one case*....*in a family of eight people*, *seven of them had* DR-TB. *When we went to check how they live*, *we saw that their house is almost like a ‘kot’ so it is sooo (sic) small*. [The Amharic word “ቖጥ” (kot) refers to traditional chicken breeding cages in Ethiopia. Those types of cages are not modern and comfortable. Instead, they are horizontally-hung sticks on which chickens stand/sleep next to each other with no or very little space among them.]*” (Shalpet*, *clinical nurse*, *FGD)*

Even though it was not clear whether the number of members in a family is problematic by itself, the mismatch between the numbers in a group of people (family) and the place or house they live in is clearly presented as problematic. Living as a big family in houses, with very limited ventilation and confined space among members, fosters the transmission of DR-TB to others if one family member is already infected. In order to determine how and where this one family member could be infected, it was necessary to explore any other indications of crowded settings than simply poor housing conditions. The participants mentioned the crowded public transport facilities as significant facilitators of DR-TB in Addis Ababa, as people with no history of TB themselves or their families can become DR-TB patients. A lack of proper ventilation in public transportation, together with a weak immune system resulting from poor nutrition, increase the chances of being infected with drug-resistant strains of TB.

“*There are patients who had no history of TB*, *in both themselves and their families*. *So from where do such people get the disease (DR-TB)*? *It could be because of public transport*.*” (Kash*, *clinical nurse)*

“*Public transport*.....*it is a biiig (sic) problem*. *There are around 20 people in one minibus that should have carried 12*.*” (Kofi*, *lab technician)*

#### Movement of clients

The participants stated that patients quit treatment because they move to other places in search of better job opportunities. TB patients normally follow their treatment through a program called directly observed treatment, short-course (DOTS) – the WHO’s internationally recommended strategy for TB control. This involves the treatment of TB patients with first-line TB drugs for a period of 6-9 months. TB patients therefore come to the TB clinics on a daily basis to take TB drugs or swallow them in the presence of a healthcare provider. This is to ensure that TB patients take the TB drugs in the required dosage and time, with the presence and supervision of a health professional. However, when patients move to another place, this becomes interrupted. Linking these patients with the health facilities available in the new areas they move to is difficult due to a lack of good health communication facilities. This facilitates drug resistance in the TB bacteria. Moreover, the DR-TB patients moving to a new place helps facilitate the spread of the DR-TB infection given the poor housing and living condition at the new place they are moving to.

“*It is to win daily bread that people move here and there*. *They (DR-TB patients) move here to this area*, *rent small rooms*, *start treatment whenever they are diagnosed with DR-TB*, *then they leave for other places*. *So*, *does this not contribute to the spread of DR-TB*?*” (Hansir*, *clinical nurse)*

### Theme 2: Experiences of management and control of DR-TB

This theme reflects the healthcare providers’ experiences in connection with the management and control of DR-TB. It is divided into three further subthemes related to experiences of the factors affecting the management of TB: public awareness of DR-TB, health-care workers’ clinical knowledge on DR-TB and cultural values among clients.

#### Public awareness of DR-TB

Participants reported that there is a lack of awareness in the community on DR-TB. Even though the media sometimes mentions “TB”, according to the participants DR-TB does not receive any attention whatsoever. They stated that patients are superficially aware of drug-susceptible TB, mostly about their transmission. On the contrary, the participants added that DR-TB, and even the very name of it, appears as a complete surprise to patients, especially when they briefly learn what the disease is all about. They reported that some DR-TB patients do not take protective measures – for example, putting on protectives masks, avoiding (unprotected) physical contact, etc. – in order to not infect family members and relatives who come to visit DR-TB patients in DR-TB wards. The participants also witnessed that the visitors themselves do not protect themselves, and make close physical contact. The healthcare providers noted that some DR-TB patients and their visitors do not take protective measures, even though they are informed of the health consequences of not doing so. Although a lack of awareness was reported as a major factor for this, there is also another contributing factor, which is further discussed under the subtheme “cultural values among clients.” Lastly, the participants also indicated that people confuse the symptoms of DR-TB with that of, for example, the flu, and do not seek medical treatment, which allows the disease to stay in the community undiagnosed and to infect others.

“*People know nothing about the disease until they become patients and are admitted here*.*” (Almet*, *lab technician)*

“*Our community lacks awareness*. *People say ‘this is just the flu’ and do not go to healthcare centers*.*” (Kasge*, *health officer)*

#### Healthcare workers’ clinical knowledge on DR-TB

The participants noted a lack of clinical knowledge among healthcare providers on DR-TB. In most cases, healthcare providers join the TB care workforce with little or no knowledge of TB in general. With regard to DR-TB, most of the participants mentioned that they had not heard of the term “DR-TB” until they started working with DR-TB patients. Consequently, such a knowledge gap brought an inability to take time in explaining the disease to DR-TB patients during treatment sessions. It has resulted in a lack of competence in understanding DR-TB patient experiences in connection with the disease – such as treatment side effects and complications. Some of the participants also said they received TB training that lasted for a few days (a max. of five) when they started working on DR-TB care. Nevertheless, they felt that this training was not enough, and that they learned more through work experience.

“*When we*, *the healthcare workers*, *are assigned to work on* DR-TB, *we get only a five-day training so we do not know well what we are going to work with*.*” (Alto*, *clinical nurse)*

“*Healthcare workers cannot give enough time to patients because they lack knowledge*.*” (Melsa*, *clinical nurse)*

#### Cultural values among clients

In the interviews, the participants mentioned culture as one factor responsible for the current condition of DR-TB. They claimed that cultural values that focus on sharing, intense physical contact and attachment help facilitate the spread of DR-TB. According to the healthcare workers, it is an undeniable fact that people can sometimes give more weight to cultural values than to scientific knowledge. Due to this, people have a strong attachment to others they know, and remain physically close regardless of the presence of DR-TB infection among them. Even when patients are well informed about the transmissions of DR-TB, they make unprotected physical contact with their families. As the participants expressed, this is due to cultural values. The coming together of two forces – cultural values that appreciate closeness and crowded living conditions – make the transmission of DR-TB more probable than it could be with the absence of one of these.

“*I grew up with my grandmother and I still sleep with her on the same bed*. *And I have allergies and sometimes I cough and feel sick*. *No matter how strong my cough is*, *I just sleep with my grandmother*. *I do this even though*, *as a health professional*, *I shouldn’t (smiles)*. *It is our culture*. *We love to be closer to one another*.*” (Hansir*, *clinical nurse)*

Participants also expressed that when using terms like *“bird”* and *“mich*,*”* people tend to use home-made herbal medicines and treat themselves at home. The word “bird” (”ብርድ” in Amharic) is a cultural explanation for symptoms like coughing. The word ”mich” (”ምች” in Amharic) is a culturally created disease name. The major cause of this disease is believed to be exposing one’s unclean body part to direct sunlight. Symptoms include irritation or a rash in that body part. Even though none of the healthcare workers in this study reported negative perception towards traditional medicine, some healthcare workers believe that the practice of not seeking modern healthcare services through the community prohibits an early diagnosis of DR-TB, and thus facilitates its spread.

“*It is because of our culture that we do not want to go the hospitals when we feel sick*. *We prefer to say ‘the pain goes away*, *no worries’ or ‘it is ‘bird’*.*” (Algon*, *health officer)*

## Discussion

In this study, in-depth interviews with 18 participants provided information on healthcare workers’ perceptions and experiences on the management and control of DR-TB in Addis Ababa, Ethiopia. The findings in this study show that healthcare workers perceive the current state of DR-TB in Addis Ababa as serious. Furthermore, they have a gloomy forecast for the disease, with all asserting that DR-TB will continue to be a huge and growing public health challenge for the country. Currently, there are significant numbers of both MDR and XDR tuberculosis cases being treated in the two TB specialized hospitals in Addis Ababa. As explored through this study, healthcare workers anticipate more and more MDR and XDR-TB cases in the disease’s foreseeable future. This corresponds to a recent study done in India, the Philippines, Russia and South Africa, where an increase of MDR/XDR TB is predicted [30]. The findings also show that the reasons for not controlling DR-TB are primarily non-biomedical. As noted by Mangum and Dacanay [31], the social factors of DR-TB are worth attention in order to effectively address the disease.

As opposed to the WHO’s efforts to create a healthcare utopia of a world free of TB [32], this study shows a frightening future as predicted by healthcare workers. This relates to what medical historian Christoph Gradmann argues – that the lament over absent novel antibiotics is also a manifestation of a dystopia – in which novel antibiotics are lacking but a fixation on drugs is upheld, rather than being a means to redeeming sufferers from diseases [33]. As shown in the findings, controlling DR-TB is made impossible and the absence of a silver lining to the cloud promising a better future is easy to notice. There are unaddressed social factors, health system barriers and patient conditions, which play a significant role in the epidemiology of the disease [31,34]. The prediction of a worse future in this study agrees with the conclusion by Sharma et al. [30] that the burden of MDR and XDR tuberculosis will continue to increase in India, the Philippines, Russia and South Africa.

Our study confirms that it is impossible to overcome DR-TB without addressing poverty. Among other factors, poverty is the first to take the blame for the current and future epidemiology of DR-TB. Applying the concept of “structural violence,” the experiences from Ethiopia [35] and Haiti [36] demonstrated that TB in general can be seen as a disease of poverty and therefore needs to be placed in a wider context than that of biomedicine. The concept of “structural violence” – a concept Johan Galtung coined to describe the failure to address TB [37] – illuminates the connections between TB and poverty. Elaborating on this concept, Galtung stated:

If a person died from tuberculosis in the 18th century it would be hard to conceive of this as violence since it might have been quite unavoidable, but if he dies from it today, despite all the medical resources in the world, then violence is present according to our definition. [37]

Our results tie in well with that of a study conducted in India, showing that poverty forms the context in which tuberculosis sustains itself as a serious social catastrophe in the country [38]. Depending on the healthcare workers’ experiences in our study, patients coming to the healthcare facilities are people who have nutrition problems, are socioeconomically disadvantaged and suffer from poor housing and living conditions. The public health society and health program planners should first acknowledge that the effect of structural barriers is equally important as that which takes place with a person’s biology. For instance, in the case of MDR-TB, poverty is as equally important as irregular treatment and monotherapy, since the latter are less likely to occur in well-to-do societies. Therefore, considering poverty as one cause of MDR-TB in the global south should not be tendentious. DR-TB is a disease of poverty [39], hence, social processes that define DR-TB as a socially produced biological incident should not be overlooked [36]. As medical anthropologist Paul Farmer has argued, pointing to individual patients as the major cause of the spread of DR-TB strains is losing sight of the true roots of the disease – the social forces and inequalities that can be embodied as biological events [40].

Poor nutrition has come out as one determinant defining the current and anticipated state of DR-TB. The findings indicate that poor nutrition results in treatment disruption, which – according to the WHO – plays a significant role in the mechanism by which *Mycobacterium tuberculosis* become resistant to drugs [41]. Given the heavy and toxic drugs that patients take on a daily basis, following a nutritious diet is an imperative. However, as noted by the healthcare workers, patients do not have the means to do this. In addition to this, traveling to the healthcare facilities on a daily basis is not only tiresome, but also financially disadvantageous. It results in transportation costs and losing jobs due to absence from work in order to visit healthcare facilities. This is in line with what the healthcare workers in Russia reported, namely that poor nutrition and financial problems hinder effective treatment [34]. Such barriers uncover the fact that adherence to treatments is not only a personal decision an individual makes. In this way, an adherence to treatment becomes a decision of two parties: the DR-TB patient and the large-scale structural issues. Addressing poverty is not a choice, but a necessity that policymakers and public health reforms need to take into account in their move towards the eradication of DR-TB. Defining compliance in such a way will not leave the poor as seldom understood. Therefore, our study, together with the available literature, shows that controlling DR-TB is made difficult by the existence of poverty. One specific way of understanding this clearly is giving an account to the relationship between DR-TB and crowded living. The highly crowded public transport facilities and the presence of big families in small houses are among the reasons that make the control of DR-TB unattainable. As indicated in this study, there are instances that in some families almost all were infected with DR-TB, and came to seek care at health facilities. This is consistent with a study that presents crowded living conditions as part of the patient’s social world which, together with other features, defines drug resistance in tuberculosis [42].

Our study illuminates the socioeconomic and structural facets of DR-TB. In addition to clinical DR-TB, which is caused by drug-resistant *Mycobacterium tuberculosis*, the poor and underprivileged suffer from another typology of DR-TB – “structural DR-TB.” This side of DR-TB relies on the wider factors that the individual is incapable of avoiding. “Structural DR-TB” does not give the poor patient an option to be safe from the disease; it does not depend on the individual’s choice. This echoes findings and arguments in previous studies from different parts of the world: Ethiopian TB patients who had no (monetary) power to face the costly and delayed treatment procedures [35]; a Haitian who was not capable of keeping his pharmacy safe from bombing, so that TB drugs could be available for him [36]; South African children, who did not have the chance to choose between a healthy diet and malnutrition [43]; and English people in Liverpool, who did not have the choice to live in a less crowded environment and thus reduce their vulnerability to TB [44,45]. The intention behind discussing “structural DR-TB” is not to dichotomize the infectious disease and come up with another set of epidemiological explanation. However, it is to illuminate that side of the disease which could remain unreachable by the efforts of traditional biomedicine to eradicate the old disease from our planet; it is to augment the orthodox biomedical approach to DR-TB by pushing for a more comprehensive perspective. Focusing only on the individual’s biology and behavior, and using words such as “compliance” and “infection”, would hence be overlooking equally important facets of DR-TB.

Our study showed perceived connections between mobility and crowded living in Addis Ababa, and how this contributes to the spread of DR-TB. People migrate to the city in search of better job opportunities and jam themselves into crowded living conditions. Due to this reason, the number of small houses with big or extended families increases. According to our participants, the infection of one person in such large and confined household conditions results in the infection of the entire family. Mobility is making the whole city crowded – as manifested through housing conditions and public transport – and as a result, it is negatively influencing the chances of controlling DR-TB. The existing literature shows that urban residence can be a risk factor for DR-TB, as poor and crowded living conditions characterize developing urban settings [46].

Poor public awareness is the other feature that constituted our participants’ perception towards the facilitators of DR-TB. Our study shows that limited biomedical knowledge on DR-TB among health professionals negatively affects the healthcare services they provide. This coincides with the healthcare workers in Tanzania, who reported that health workers’ lack of awareness on TB constrained effective treatment [47]. In addition, a lack of skilled healthcare workers to diagnose and manage DR-TB restrains better treatment [48]. Participants also stated that there is lack of public awareness about DR-TB, which contributes to the increase of DR-TB. Previous studies have shown that a lack of awareness about the transmission and symptoms of TB is accountable for the spread of the disease [35,49]. Our participants reported that DR-TB patients do not take measures to protect others (e.g. by wearing masks). Similarly, family members of the patients are not careful when they provide care to DR-TB patients at home. The culture of closeness and intimacy in Ethiopian societies can be seen together with a gap in awareness. This raises a question that needs further investigation: Is it due to a lack of awareness about DR-TB, or due to a lack of commitment to compromise cultural values and act according to the acquired knowledge, that causes DR-TB patients and their families to not follow protective measure? Our study sheds some light on this enquiry, exploring the connections between cultural values and DR-TB.

Our findings identified culture as determining DR-TB in two ways. Firstly, a culture of physical and emotional intimacy among people in Ethiopia fosters transmission of the disease. Notwithstanding that a lack of awareness about DR-TB by itself can be seen as a factor, our study also shows that in the face of strong conformity to one’s cultural values, the presence of DR-TB awareness does not help by itself. Therefore, it would be correct to conclude that acquired medical knowledge fails to function if some account is not given to the (cultural) context of the knowledge host. Secondly, lacking the habit of seeking medical care is the other example our participants used to elaborate on the relationship between culture and DR-TB. Our participants stated that patients and the wider community generally give healthcare facilities the least priority due to cultural values, which do not encourage seeking modern medicine. Consequently, there is a higher probability for people infected with DR-TB to stay at home, thereby confusing the symptoms with other less serious diseases such as the flu. In an earlier study in Ethiopia, it is demonstrated that in the Ethiopian community when using words such as “bird,” TB patients confuse TB symptoms and do not seriously take an adherence to TB treatments [35].

This study has some limitations. There might be a selection bias, as the participants were purposively selected. Since all of the interviews were conducted during work hours, some participants could not finish the interviews without being interrupted by work duties. In this case, the interviewer paused the discussions until the participants came back, which might have affected the coherence in the discussions. Some TB wards were very busy, and were sometimes closed due to meetings. Some of the participants were also recruited by medical directors at the healthcare facilities. This might have influenced the participants’ decision, since there were hierarchical relations between the medical directors and the healthcare workers. In this case, the principles of informed consent and voluntary participation were explained in detail to both the medical directors and the participants.

## Conclusion

This qualitative study has explored that healthcare workers perceive DR-TB as a continuously increasing and catastrophic public health issue. This goes against the WHO’s estimation and target. The perspectives drawn from the healthcare workers helped to shed more light on the image of DR-TB in a developing country context. The study has shown that DR-TB is not simply confined to the sphere of biomedicine. An exclusive focus on the biomedical facets of DR-TB can draw attention away from targeting a key issue – the connections between health-related problems and socioeconomic and cultural forces. Taking a step back from such a singular focus can help health authorities and decision-makers critically think about the wider spheres of local realities in connection with DR-TB. As the current study indicates, the incapability to acknowledge and redress factors that perpetuate the disease – such as poverty, the quality of governance, culture, healthcare workers and a general public awareness of DR-TB – makes TB control efforts vain. These factors not only contribute to the problem independently. They are also connected to each other, thereby making the management and control of DR-TB more difficult, and predicting a dystopia in the epidemiology of the disease. Tragically, in the face of the overwhelming support for massive invention and the production of antibiotics and chemotherapies, together with context-insensitive and burdensome treatment procedures, it is a challenge to focus on the wider social forces and inequalities.

A future study that brings perspectives from different study populations – DR-TB specialists, patients and TB program representatives – could provide further insights on the topic. Moreover, we suggest conducting studies that investigate existing gaps in current TB control efforts, and recommend strategies to fill the gaps. Additionally, there should be efforts, through both training and health education, to increase knowledge and awareness among healthcare workers and communities about DR-TB.

## Supporting information

S1 File Data collection tool. (DOCX)
