# Supplementary material for: Exploring local realities: Perceptions and experiences of healthcare workers on the management and control of drug-resistant tuberculosis in Addis Ababa, Ethiopia
Source: PLoS One. 2019 Nov 13;14(11):e0224277. doi: 10.1371/journal.pone.0224277 (PMC6853283; doi:10.1371/journal.pone.0224277)

# Annexure 1: Data collection tools

## In-depth interview guide

The interview questions are semi-structured, meaning that they are neither unstructured conversations nor a list of closed questions. The questions are therefore general designed to hold the interview around the research idea. By using open-ended questions and probing, the researchers plans to go deep and wide to get comprehensive and detailed information.

Demographics:

Can you give a brief introduction of yourself?

Work environment:

How do you describe your work environment?

In what functions are you working on TB?

What do you think are your important roles/responsibilities in working on TB?

Perspective on DR-TB:

When did you first hear about drug-resistant TB? How do you explain your feeling at that time?

How this knowledge did form what you learned later?

Why do you think TB bacteria become drug resistant?

What are the significant sources of information about DR-TB for you?

How is DR-TB affecting your daily work?

How do you imagine about the future of DR-TB in Addis Ababa?

Do you have anything to add?

## Focus group discussion guide

How do you describe DR-TB?

How relevant is the scientific medical knowledge to health workers for their practice with DR-TB patients?

What life aspects are affected as a result of being a DR-TB patient?

How is DR-TB affecting your work environment?

According to you, what roles do health workers have in the development of DR-TB?

Do you have anything to add?

# Translated in-depth interview and FGD guides in local language (Amharic)


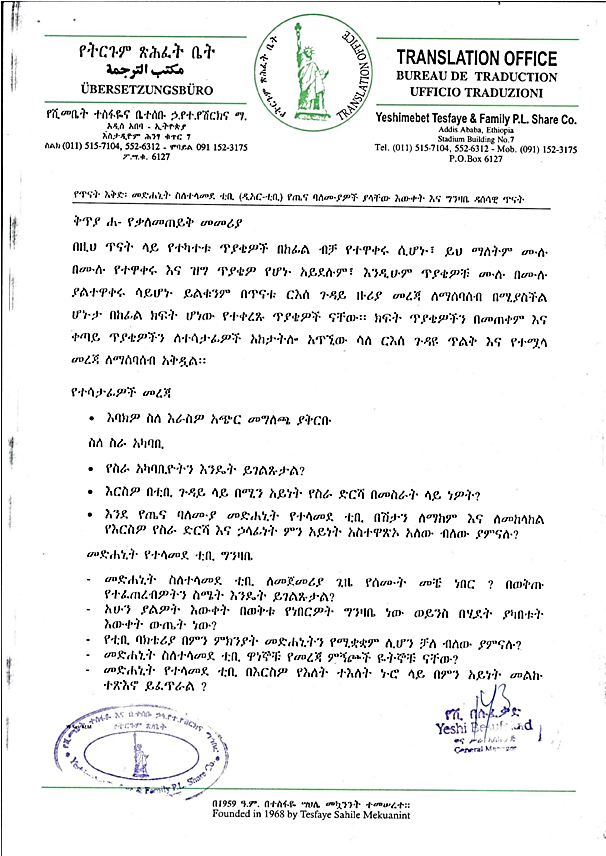


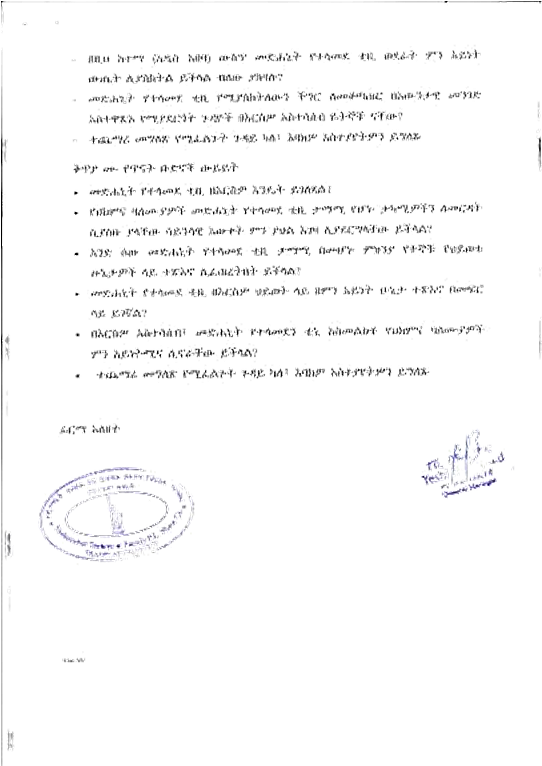

Supplement: S1 File — (DOCX) [file pone.0224277.s001.docx]
